# Development of placental abnormalities in location and anatomy

**DOI:** 10.1111/aogs.13834

**Published:** 2020-03-18

**Authors:** Charlotte H. J. R. Jansen, Arnoud W. Kastelein, C. Emily Kleinrouweler, Elisabeth Van Leeuwen, Kees H. De Jong, Eva Pajkrt, Cornelis J. F. Van Noorden

**Affiliations:** ^1^ Department of Obstetrics and Gynecology Academic Medical Center Amsterdam UMC University of Amsterdam Amsterdam the Netherlands; ^2^ Department of Medical Biology Cancer Center Amsterdam Academic Medical Center Amsterdam UMC University of Amsterdam Amsterdam the Netherlands; ^3^ Department of Genetic Toxicology and Tumor Biology National Institute of Biology Ljubljana Slovenia

**Keywords:** abnormally invasive placenta, low‐lying placenta, placenta, placenta previa, placental abnormalities

## Abstract

Low‐lying placentas, placenta previa and abnormally invasive placentas are the most frequently occurring placental abnormalities in location and anatomy. These conditions can have serious consequences for mother and fetus mainly due to excessive blood loss before, during or after delivery. The incidence of such abnormalities is increasing, but treatment options and preventive strategies are limited. Therefore, it is crucial to understand the etiology of placental abnormalities in location and anatomy. Placental formation already starts at implantation and therefore disorders during implantation may cause these abnormalities. Understanding of the normal placental structure and development is essential to comprehend the etiology of placental abnormalities in location and anatomy, to diagnose the affected women and to guide future research for treatment and preventive strategies. We reviewed the literature on the structure and development of the normal placenta and the placental development resulting in low‐lying placentas, placenta previa and abnormally invasive placentas.

**Abbreviations**
dNK: decidual natural killer
MMP: matrix metalloprotease

**Key message**

Understanding the etiology of low‐positioned placenta and abnormally invasive placentas is crucial for healthcare professionals. Disorders during the apposition, adhesion and invasion phases of the implantation may cause these placental abnormalities in location and anatomy.

## 1 INTRODUCTION

The placenta is crucial for pregnancy. As the largest fetal organ, it has indispensable functions in the development and protection of the fetus.^1^

Placental abnormalities with respect to location and anatomy in pregnancy include low‐lying placentas, placenta previa and abnormally invasive placentas.^2^ These conditions form a risk of antepartum, intrapartum and postpartum hemorrhage. In addition, they can affect placental functions and interfere with maternal or fetal well‐being.^3^, ^4^ The etiology of these abnormalities is not well understood and their incidence is increasing, predominantly caused by the rising cesarean section rate.^2^, ^5^, ^6^ Other factors that affect the incidence are prior uterine surgeries or curettage, maternal age and multiparity.^2^ In addition, the incidence of a low‐lying placenta and placenta previa is increased due to endometriosis, smoking, previous placenta previa and assisted reproductive technology.^5^, ^7^, ^8^ For abnormally invasive placentas, the additional risk factor is having a placenta previa or having Asherman’s syndrome.^9^, ^10^ Treatment options are scarce and usually result in a cesarean delivery, increasing yet again the incidence of placental abnormalities in future pregnancies. To date, adequate preventive strategies, other than preventing uterine surgery such as cesarean section and dilatation and curettage, and avoiding unnecessary assisted reproductive technologies, are not available.

The placenta is part of the pregnancy from the moment that the embryo consists of a few cells until it is discharged after childbirth. As the placental formation already starts at implantation, at which point the embryo invades the endometrial wall, disorders during implantation may cause placental abnormalities in location and anatomy.^11^ Understanding of the normal placental structure and normal placental development during implantation is essential to comprehend the etiology of placental abnormalities in location and anatomy, to diagnose affected women and to guide future research in the prevention of these abnormalities. Therefore, we provide an overview of the literature on the structure of the placenta and the placental development during implantation. In addition, placental anatomical and developmental disorders are discussed.

## 2 PLACENTAL STRUCTURE

**Figure 1:**
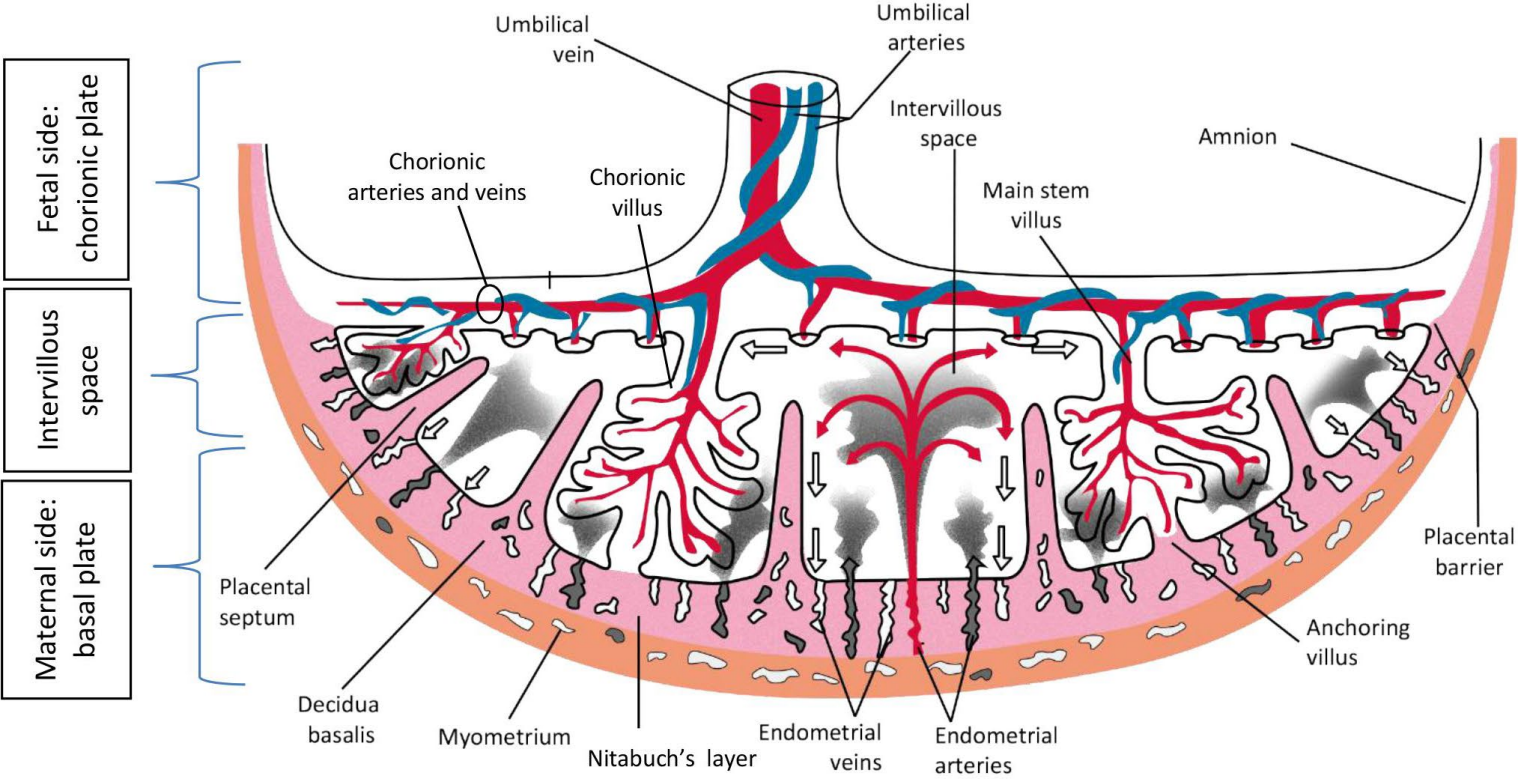
Schematic drawing of the fetal side and maternal side of the placenta in the second half of pregnancy. Fetal side: Chorionic plate that contains the amnion and main stem villi (chorionic villi). Maternal side: Basal plate that contains placental septa and decidua basalis. Red, fetal veins: Umbilical vein, chorionic veins and venules; maternal arteries: endometrial arteries. Blue, fetal arteries: Umbilical arteries, chorionic arteries and arterioles. Pink, decidua basalis, Nitabuch’s layer, placental septa. Brown, myometrium

The placenta is composed of the chorionic plate on the fetal side and the basal plate on the maternal side. The fetal side and maternal side are separated by the intervillous space (Figure 1).^12^ The chorionic plate is a thick mass of connective tissue and contains the amnion, main stem villi and the chorionic arteries and veins, which are ramifications of the umbilical arteries and umbilical vein. The chorionic arteries and veins ramify into the arterioles and venules of the main stem villi. The main stem villi project into the intervillous space and are connected to the maternal basal plate by anchoring villi (Figure 1).^13^

The basal plate is composed of a heterogeneous mixture of trophoblastic cells and decidual cells and contains the decidua basalis. In the third trimester of pregnancy, Nitabuch’s layer develops. This is the specific area from where the placenta detaches itself from the uterus at birth. From the basal plate, placental septa bulge into the intervillous space, creating a system of grooves which delimit 10‐40 elevated areas, also known as cotyledons or maternal lobes.^14^, ^15^ The basal plate is penetrated by endometrial arteries and venules. The exchange between fetal and maternal circulatory systems occurs between the main stem villi and the maternal endometrial arteries and venules in the intervillous space (Figure 1).^12^

## 3 PLACENTAL DEVELOPMENT

Fertilization is a course of coordinated events involving sperm preparation, sperm‐to‐egg binding, and fusion and activation of the fertilized egg.^16^ After ovulation, the oocyte is surrounded by the zona pellucida and the corona radiata. The sperm penetrates both layers causing a calcium wave throughout the cytoplasm of the oocyte.^17^ Due to the calcium wave, a rapid activation of glucose‐6‐phosphate dehydrogenase (G6PDH) occurs and large quantities of the reduced co‐enzyme NADPH are immediately produced. This is used as a substrate for a peroxidase enzyme, which instantly catalyzes the hardening of the zona pellucida, preventing polyspermy and thus lethal paternal triploidy.^18^ After fertilization, a diploid embryo is formed, which is the beginning of the fetus and its placenta. While it undergoes cell divisions, the embryo is passively transported towards the uterus. Around day 5, the embryonic cells are freed from the zona pellucida and the blastocyst is formed, ready for implantation.^19^ At implantation, 7‐12 days after ovulation, the blastocyst contains the blastocyst cavity, the inner cell mass or the embryoblast and the trophoblast at the periphery. The latter becomes the placenta.

Implantation is a highly organized process which involves complex interactions between the activated blastocyst and the receptive uterus.^20^, ^21^, ^22^ Implantation can be defined as “the process by which the embryo attaches to the endometrial surface of the uterus and invades the epithelium and then the maternal circulation to form the placenta”.^18^ The limited period of time during which the uterine receptivity for implantation is optimal is called the “window of implantation”.^23^ Within this window, adequate modifications of the blastocyst and endometrium create a uterine environment that is favorable for the development of the embryo and is immunologically tolerant for the semi‐allogenic graft.^23^

For implantation, complex interactions between endometrium and embryo are essential. Synchronous development of endometrium and embryo that is competent to implant is required. The implantation process consists of “apposition”, “adhesion” and “invasion”.^12^, ^20^

Dysfunction in apposition, adhesion and invasion may result in abnormal placentation, which can affect the placental architecture as well as the placental shape. Both can have long‐term clinical consequences with impaired placental function. This is associated with maternal and fetal complications such as preeclampsia and intrauterine growth restriction, which are reflected in the placenta both macroscopically and microscopically.^24^ Women with preeclampsia have defective remodeling of the spiral arteries and can have a placental shape that is more oval than round, with a reduced surface area, whereas in intrauterine growth restriction the umbilical cord is inserted into the placental margin or the fetal membranes rather than into the main placental mass.^25^, ^26^, ^27^, ^28^ Dysfunction in apposition and adhesion may result in placental abnormalities in location such as low‐lying placentas and placenta previa, whereas dysfunctional invasion may result in abnormally invasive placentas, as will be discussed below.

### 3.1 Apposition and adhesion

**Figure 2:**
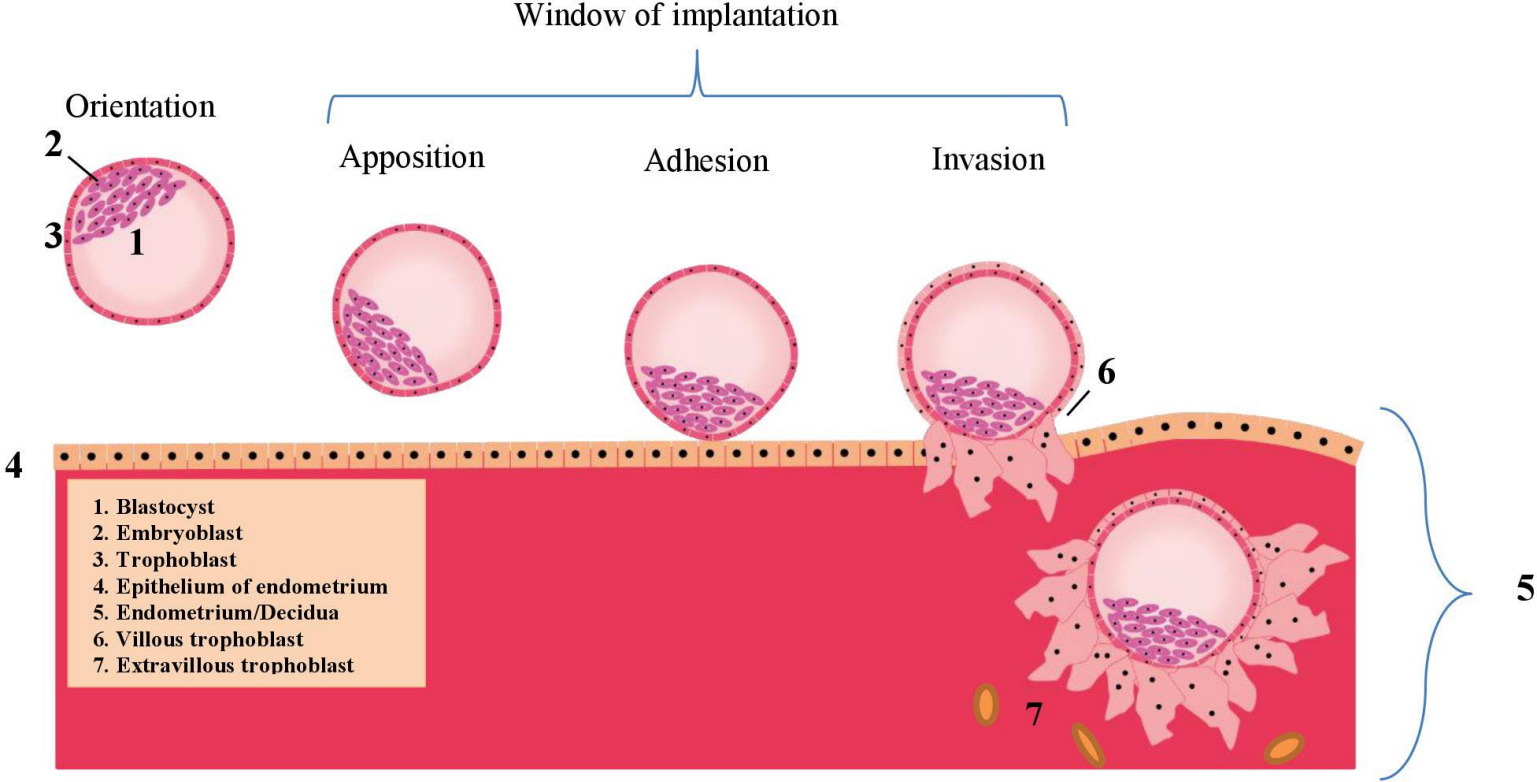
Orientation, apposition, adhesion and invasion (the window of implantation) during blastocyst implantation and the first steps in the development of the placenta

The initial contact between blastocyst and uterine endometrium is made during apposition (Figure 2). This contact determines the implantation site, which is usually the upper part of the uterus.^29^ The contact becomes tighter during the adhesion process. During apposition and adhesion, the blastocyst differentiates and an inner cell mass (the embryo) and trophoblast (the placenta) develop (Figure 2). In the same period, cells in the surrounding endometrial stroma transform to accommodate embryonal growth and invasion. This transformation of endometrial stromal cells into specialized secretory cells is called decidualization.^30^, ^31^, ^32^ The decidua is endometrium that is specialized for pregnancy. Directly beneath the implantation site of the blastocyst, it is called the decidua basalis (Figure 1) and this is the location where the placenta will develop from the invading trophoblast.

Before apposition and adhesion, the blastocyst orientates in the uterus and selects the site of implantation. For endometrial growth, embryonic growth and placentation, blood flow at the implantation site and angiogenesis are necessary. The fundus has the highest endometrial tissue blood flow and may therefore be the favorable site for implantation.^33^ The majority of embryos (76%) migrate towards the fundus, whereas a smaller proportion do not migrate and implant at the transplantation site (12%), or migrate towards the cervix (11%).^34^ After embryo transplantation near the fundus, 94% of the embryos will not migrate and implant in the fundal region.^35^, ^36^ However, it is not known whether embryos implant selectively at the endometrial site with the highest blood flow, or that embryos implanted at the site with the highest blood flow are embryos that survive. If the embryo does not implant in the area with the optimal endometrium (placental location disorders), several factors may have played a role. Placental hypertrophy increases the likelihood of the placenta lying near to or over the internal os of the cervix. Placental hypertrophy can be caused by carbon monoxide‐induced hypoxia from maternal cigarette smoking and is observed in multiple pregnancies, in multiparous women and older women.^37^

In women after assisted reproductive technology, as well as in women with endometriosis, the frequency and amplitude of uterine contractions in the implantation period are enhanced. This may cause abnormal uterine peristalsis, leading to abnormal embryo implantation near the cervical os, resulting in a lower implantation of the placenta.^38^, ^39^, ^40^, ^41^ The presence of a cesarean scar may also alter myometrial contractility, disrupting the contraction waves during implantation. Women with a previous cesarean delivery are significantly less likely to have a fundal placenta and more likely to have a placenta located at the posterior side of the uterus. So, the altered myometrial contractility and disrupted contraction waves in the endometrium after a previous cesarean delivery can cause a different or lower location of implantation, that is, a low‐lying placenta or placenta previa. Placental implantation may also occur in the area of or in a cesarean scar. In those cases, proteins that enhance endometrial receptivity during normal implantation, such as integrin β3 and leukemia‐inhibitory factor, seem to be overexpressed in that area as compared with the remainder of the uterine cavity, causing the implantation to occur in or around the scar instead of in healthy endometrium.^42^ Because there is no blood flow in a cesarean scar, the scar tends to be hypoxic, but since hypoxia stimulates trophoblast cells to proliferate, the early embryo can still develop here.^43^ Macklin et al compared the physiological hypoxia in trophoblasts and placenta with the pathophysiological hypoxia in tumors both driving expression of hypoxia‐inducible factor‐1α and ‐2α. This in turn upregulates cellular proliferation, reduces cell death and stimulates vascular remodeling, invasion in local tissues and immune tolerance.^44^ Moreover, implantation requires an environment rich in collagen. As a uterine scar is rich in collagen, the trophoblast can adhere to the uterine scar, leading to a placenta previa or low‐lying placenta.^45^ In addition to the above‐mentioned, during apposition, the blastocyst may be guided to the final implantation site by soluble mediators.^46^ Chemokines and cytokines are known for their function in leukocyte migration, and may well function in a similar manner in blastocyst migration towards and through the endometrium during implantation. Various chemokine receptors have been identified on blastocyst and trophoblast cells.^46^, ^47^, ^48^ The location of implantation is affected by local levels of mediators in the endometrium.

We conclude that for a normal localization of implantation, at least the following conditions are favorable:

- A proper endometrial environment with a high endometrial tissue blood flow,
- An endometrium without overly enhanced uterine contractions,
- A uterus without a scar,
- A non‐hypertrophic placenta, and
- Adhesion of the blastocyst in the upper portion of the uterus facilitated by mediators.

### 3.2 Invasion

During invasion, the trophoblast cells of the blastocyst differentiate into villous and extravillous trophoblasts (Figure 2). The extravillous trophoblasts are involved in invasion and either become endovascular trophoblasts, which invade into the maternal blood vessels, or interstitial trophoblasts, which migrate through the decidua and myometrium to assist vascular remodeling (Figure 2).^49^ The trophoblast invasion becomes pathological in the case of direct attachment of the chorionic villi to the myometrium instead of in the decidua. Normally, under hypoxic conditions, the cytotrophoblast cells invade the endometrium, reaching for the spiral or maternal arteries, and then differentiate into a vascular phenotype. Trophoblasts implanting in an avascular scar, may invade deeper into the uterine wall. This is caused by the absence of underlying tissue with normal vasculature and high oxygen tensions, which induce a prolonged maintenance of the invasive trophoblast phenotype and thus a prolonged invasion.^43^ Moreover, at the location of the scar, a defect of the interface between the endometrium and myometrium leads to failure of normal decidualization in the area of a uterine scar, which allows abnormally deep placental anchoring villi and trophoblast invasion.^10^ When the decidua and thus Nitabuch’s layer (Figure 1) are absent due to a uterine scar, the placental villi attach to smooth muscle fibers rather than to decidual cells. Due to decreased decidualization, the anti‐invasive factors normally secreted by the decidua, are deficient.^50^ Trophoblast invasion is a proteolysis‐driven process in which matrix metalloprotease‐2 (MMP‐2) and MMP‐9 (previously known as gelatinases A and B, respectively) play a major role.^51^, ^52^, ^53^ Inactive pro‐ or pre‐pro forms of MMP‐2 and MMP‐9 are activated by pro‐invasive factors that initially are produced by, among others, natural killer cells and in later stages, decidual cells. In the case of an MMP‐2 or MMP‐9 deficiency, trophoblast invasion is compromised, for example in preeclampsia due to MMP‐9 deficiency in the embryo.^52^ Normally, when trophoblast invasion has been completed, decidual cells inhibit MMP‐2 and MMP‐9 activity by the release of anti‐invasive factors such as protease inhibitors.^50^ Decidual natural killer (dNK) cells play a crucial role in regulation of trophoblast invasion, by controlling the function of the extravillous trophoblasts.^54^, ^55^, ^56^ The balanced interactions between the dNK cells and the extravillous trophoblasts result in a controlled placental invasion.^57^ NK cells are traditionally known as killer cells in tumors and microbial infections but in the last decades appear to be immunomodulatory cells as well.^54^, ^55^, ^56^ dNK cells are poorly cytotoxic but are major producers of cytokines, growth factor and angiogenic factors and facilitate immune tolerance, implantation, trophoblast invasion and vascular remodeling to ensure successful pregnancy. dNK cells are also known as uterine NK (uNK) cells or endometrial NK (eNK) cells.^58^ Trophoblast invasion has functional similarities with angiogenesis, invasion and metastasis of cancer.^53^, ^59^, ^60^ In particular, endogenous inhibitors and interactions with protease‐binding macromolecules such as heparin and α2‐macroglobulin limit the highly destructive proteolytic power at the posttranslational level. 61, 62, 63, 64 This complex balance between proteases and protease inhibitors was a major cause of the failure of many clinical trials of synthetic protease inhibitors as anti‐cancer therapy.^41^ The tight control of protease activity is likely lost as well in cases of abnormally invasive placentas.^62^

We conclude that failure of normal decidualization due to uterine scarring results in pathological trophoblast invasion. Deviant proteolytic activity and/or a disturbed release of protease inhibitors by the decidua may also result in pathological trophoblast invasion.

## 4 PLACENTAL ABNORMALITIES

### 4.1 Low‐lying placentas and placenta previa

**Figure 3:**
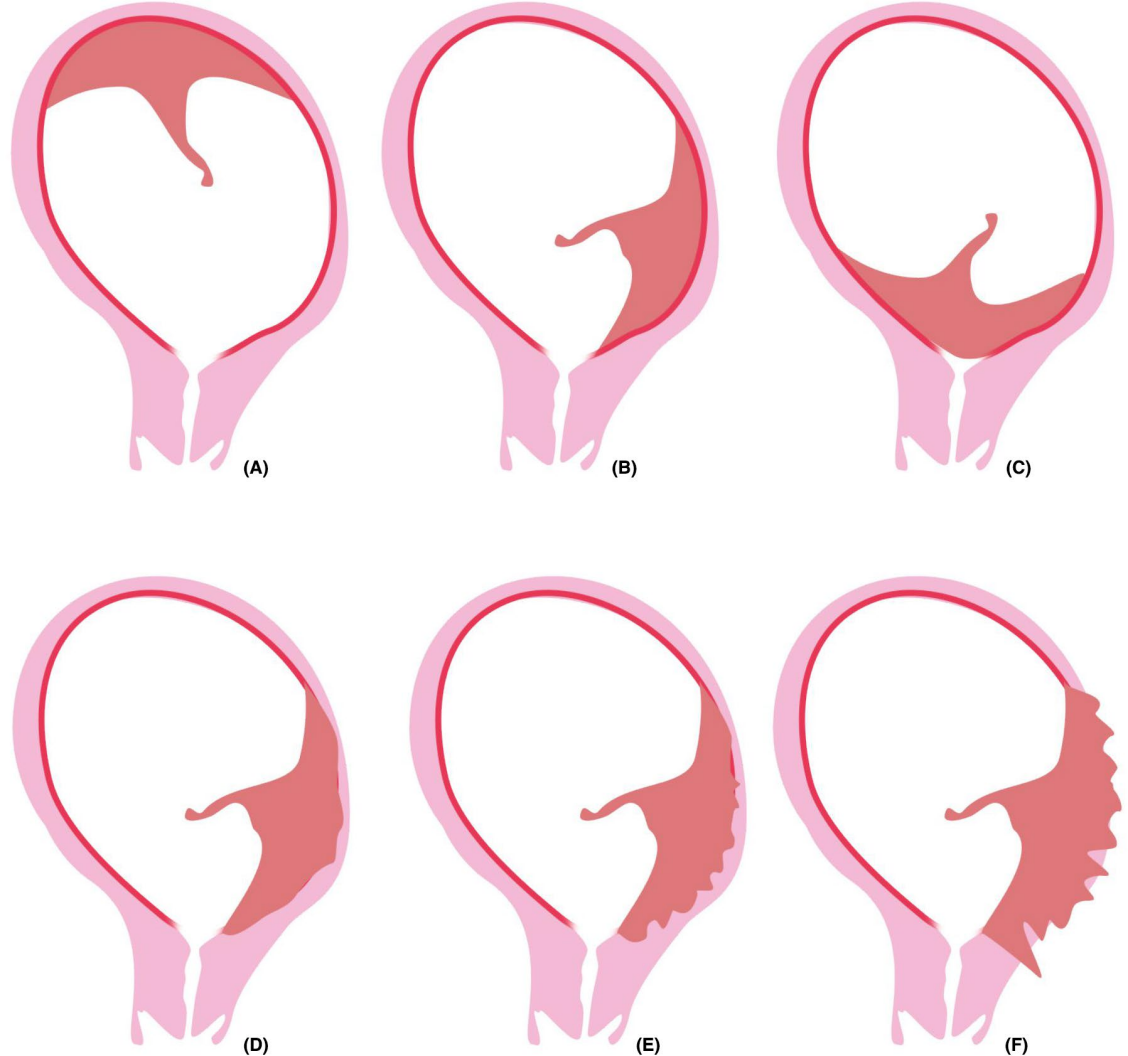
Placental abnormalities in location and anatomy: (A) normal localization, (B) low‐lying placenta, (C) placenta previa, (D) placenta accreta, (E) placenta increta, (F) placenta percreta

Low‐lying placentas and placenta previa, here defined as low‐positioned placentas, are located in the lower uterine segment (Figure 3B‐F). The definition of a low‐positioned placenta comprises two entities: low‐lying placenta with an edge of the placenta near to (<20 mm) but not overlying the internal os of the cervix (Figure 3B) and placenta previa that completely covers the internal os of the cervix (Figure 3C).^65^ Low‐positioned placentas are associated with increased obstetric risks due to excessive blood loss in the 3rd trimester and during delivery.^66^, ^67^, ^68^ Women with placenta previa require cesarean delivery because of the risk of excessive blood loss. Asymptomatic women with a low‐lying placenta have no strict contraindications for a trial of labor in a clinical setting but do have a higher risk of blood loss.^69^ One of the major adverse neonatal outcomes associated with placenta previa is preterm delivery, as the preterm delivery rate is 26.9% for women with a low‐lying placenta and 43.5% for women with a placenta previa. A cesarean section before the scheduled delivery date is performed in 43% of women. Of those, 46% have an emergency delivery before 37 weeks and in 22% even before 32 weeks.^38^, ^70^, ^71^, ^72^, ^73^ Low‐lying placentas and placenta previa detach more easily from the underlying basal plate due to reduced blood flow in the lower uterine segment. A vicious cycle consisting of placental detachment, vaginal bleeding, cervical shortening, cervical dilation and contractions is considered to be responsible for the increased risk of preterm birth.^67^, ^74^ Because of the high risk of complications in the case of a low‐lying placenta or placenta previa, the diagnosis is important and is usually made by ultrasound in the second trimester. A low‐positioned placenta occurs in 5% of all women in the second trimester. However, not all second trimester low‐positioned placentas remain low‐positioned. Due to a phenomenon called “placental migration”, in which the placenta migrates upwards during pregnancy, the incidence of low‐positioned placentas decreases to .3%‐.9% in the third trimester.^65^ Thus, over 90% of women with a low‐positioned placenta in the second trimester are not at risk in the third trimester.^75^, ^76^ Placental migration can occur due to trophotropism or dynamic placentation. Trophotropism is the process of atrophy of thin placental margins due to poor vascular supply. The isthmic portion between the body and cervix of the uterus develops into the lower uterine segment. This part of the uterus has a thinner muscular wall with less vasculature. The uterine body has a thick muscular wall and more abundant vascular supply, thus as pregnancy continues, the placenta migrates to the upper portion of the uterus with more abundant vascular supply. Trophotropism also explains why placenta previa migrates less often than low‐lying placentas and why anterior placentas migrate more often than posterior placentas. In the case of a placenta previa, overlying the internal os of the cervix and being implanted in the cervical area, the cervix establishes an improved blood supply so atrophy of the placental margin is less likely to occur.^77^ Anterior placentas migrate more often than posterior placentas.^75^, ^76^ This is probably due to the trophotropism. The anterior lower uterine segment is usually much thinner and consequently has less blood supply than the anterior uterine body. This results in an upward migration of the placenta as discussed above. However, the posterior lower uterine segment is usually less thin compared with the posterior body, resulting in little or no migration. This results in less migration of posterior located low‐lying placentas. Another explanation is dynamic placentation, in which the anterior uterine wall expands more than the posterior wall as the uterus grows. The lower uterine segment in particular becomes larger during pregnancy due to elongation and hypertrophy, causing enlargement of the uterus mainly at the anterior side, explaining placental migration, especially in anterior placentas.^78^ Due to the mentioned trophotropism of placental tissue, placenta previa in the second trimester is an important risk factor for vasa previa.^72^ Vasa previa is a complication of pregnancy in which the fetal blood vessels lie outside the chorionic plate. The vessels lie within the membranes and specifically overlying the cervical os.^79^ In the case of a second trimester placenta previa, the atrophy causes placental tissue overlying the internal os to vanish, the vessels may persist and vasa previa can appear. This can also be the case for a placenta not overlying the internal os of the cervix with a velamentous cord insertion. In that case, the vessels between the insertion of the umbilical cord and the placenta lie within the membranes overlying the internal os of the cervix. Therefore, vasa previa should always be ruled out with transvaginal ultrasonography in the third trimester in case of a second trimester placenta previa.^79^

### 4.2 Abnormally invasive placentas

Abnormally invasive placentas are characterized by abnormal trophoblast invasion into the uterine wall and direct contact of villous tissue with the underlying myometrium, without a decidua in between. This causes failure of placental separation at delivery followed by subsequent bleeding.^12^, ^80^ Abnormally invasive placentas are classified according to the depth of placental invasion (Figure 3D‐F). In placenta accreta, the placenta is in direct contact with the myometrium (75%); in placenta increta, the placenta invades into the myometrium (18%); and in placenta percreta, the placental invasion extends beyond the uterine serosa and into surrounding structures such as the bladder (7%).^80^, ^81^ The incidence varies from 1 in 533‐70 000 deliveries, depending on the definition, study population and study period. The incidence is rising, which can be attributed to the increasing rate of cesarean deliveries worldwide.^80^, ^82^, ^83^, ^84^ The incidence of abnormally invasive placentas increases with the number of previous cesarean deliveries. Maternal morbidity and mortality can occur because of severe and sometimes life‐threatening hemorrhage. Normally, separation of the placenta from the uterine wall occurs at the decidualized endometrial stroma between the contracting myometrium and the non‐contracting placenta, Nitabuch’s layer, a layer that is formed during the third trimester of pregnancy. However, the absence of a decidua that prevents separation causes a clinically adherent placenta and subsequent bleeding.^80^ Whether the increased risk for retained placenta in women with a previous cesarean delivery is based on the same mechanism as in abnormally invasive placentas is debatable. The risk of having a retained placenta is increased in women with a previous cesarean delivery and this risk is particularly high for women with a placenta previa.^80^, ^81^ Thus, one can assume that a retained placenta in women with a previous cesarean delivery shares the same mechanism as in abnormally invasive placentas, although the invasiveness is less extensive. On the other hand, there is no difference in retained placentas for anterior placenta previa (located at the side of the cesarean scar) and posterior placenta previa.^81^ In addition, there are no differences in myometrial thickness, an ultrasonographic marker for abnormally invasive placentas, in women with and women without retained placentas and a previous cesarean delivery, showing the ambiguities considering this matter.

Ideally, abnormally invasive placentas are diagnosed antepartum, since it may lead to massive hemorrhage that requires emergency peripartum hysterectomy. Antepartum diagnosis with the use of sonography has a sensitivity of 77%‐87% and a specificity of 96%‐98%.^85^ The abnormally invasive placenta is, just as low‐lying placentas and placenta previa, usually diagnosed in the second trimester. However, sonographic identification of abnormally invasive placentas is already possible in the first trimester, which enables early diagnosis, as some characteristics can then already be detected.^38^, ^86^ The diagnosis of an abnormally invasive placenta is made by ultrasound, sometimes in combination with magnetic resonance imaging (MRI).^87^, ^88^ Timely diagnosis enables adequate obstetric management in a multidisciplinary setting, thus allowing a planned cesarean hysterectomy without placental delivery. Imaging techniques have advanced over the years but the depth of invasion and thus the definite diagnosis can only be established by histopathological inspection of a tissue sample obtained during hysterectomy. The diagnosis is made when the chorionic villi are embedded in the myometrium in the absence of a decidual layer.^89^, ^90^ However, the histopathological diagnosis may not always be the gold standard, as myometrial fibers can also be found in the basal plate of normal placentas, or the pathological specimen cannot be evaluated in case of a severely damaged uterus with placenta percreta, or cannot be obtained in case of conservative management.^90^ Since fertility cannot be preserved with this approach, an alternative option is a cesarean delivery, combined with aortic balloon occlusion, followed by uterine artery embolization.^91^, ^92^

The best measure to prevent an abnormally invasive placenta is to prevent a scarred uterus, thus to prevent a first cesarean section or prevent dilation and curettage in unwanted pregnancies or miscarriages.^93^ A cesarean section cannot always be avoided, but then its timing is an important factor. The Nordic Obstetric Surveillance Study (NOSS) reported a relative risk of 4.1 (95% CI 2.0‐8.1) of having an invasive placenta after a first elective cesarean delivery compared with an emergency cesarean delivery.^94^ Another recent single‐center, case‐control study in which 65 cases with a placenta previa accreta and 102 controls matched for placenta previa were included, showed that women with a primary elective cesarean delivery without labor were more likely to develop an invasive placenta in the subsequent pregnancy than were women undergoing an emergency cesarean delivery (odds ratio [OR] 3.0, 95% CI 1.5‐6.1). In line with these results, another recent study reported that a prior cesarean delivery without labor is associated with a twofold increase in odds for abnormally invasive placentas in a subsequent pregnancy, compared with women with a cesarean delivery during labor. It was suggested in this study that the chance of an abnormally invasive placenta is increased due to disruption of Nitabuch’s layer between the placenta and the myometrium, due to the incision of the cesarean section.^95^ In contrast, only one recently performed retrospective cohort study analyzing 207 women with a placenta accreta and a history of one cesarean delivery did not find any differences between a placenta accreta in women with a history of an unplanned cesarean section up to 10 cm, an unplanned cesarean section at 10 cm or an elective cesarean section.^96^

In line with these latter results, it is hypothesized that a cesarean delivery before the morphological and immunological changes which are associated with uterine activation of labor may result in increased uterine damage. The higher risk of an invasive placenta in the subsequent pregnancy is caused by performing a cesarean section into a thick uterus without labor, in contrast to an incision through a thinned myometrium during labor.^97^

**Figure 4:**
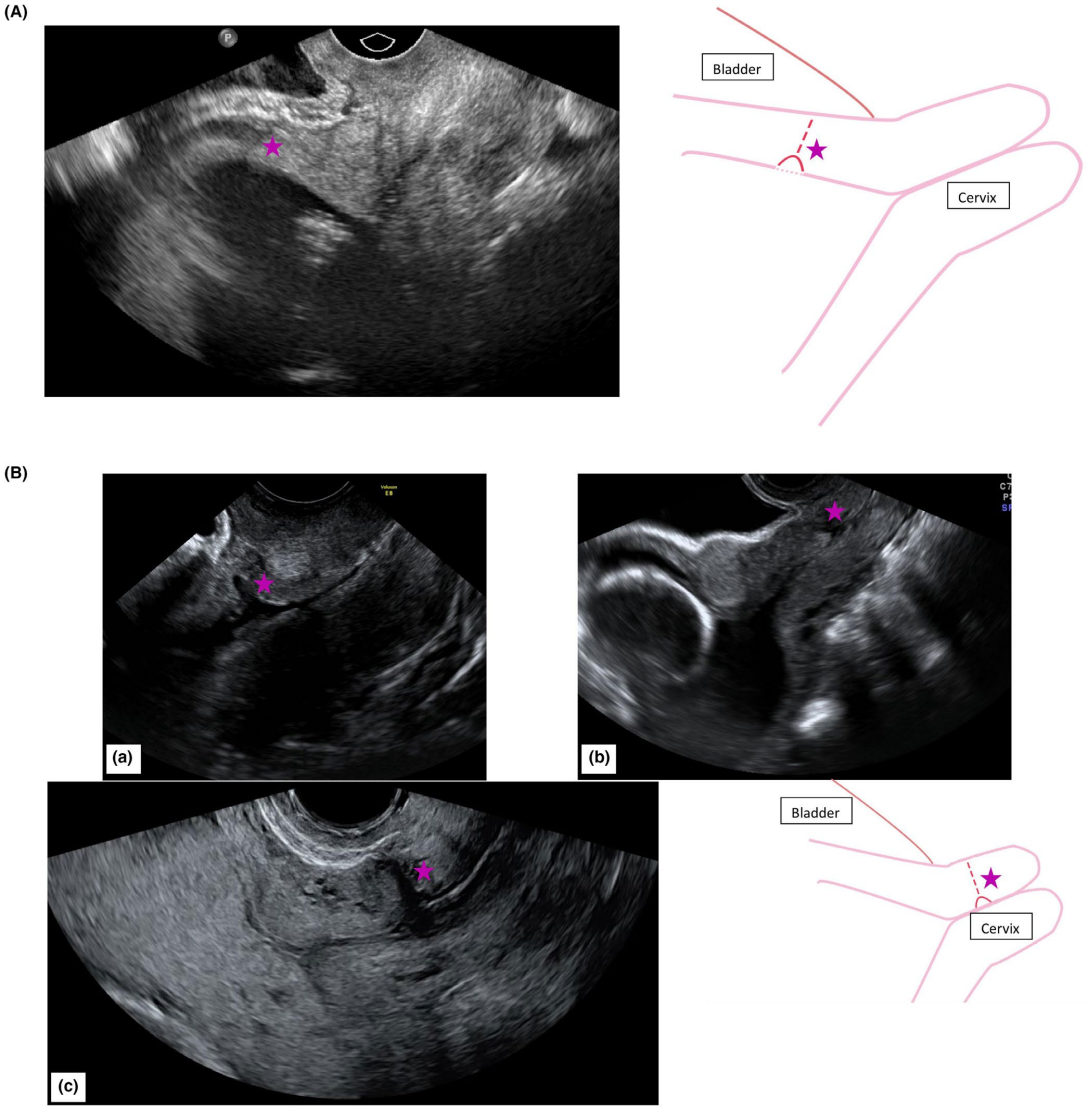
(A) The cesarean scar is intrauterine in a women at 20 weeks of gestational age after having a planned cesarean delivery without any contractions or effaced cervix, as her baby was laying in breech position. (B) Vaginal ultrasound in three different women of (a) 13 weeks of gestational age, (b) 20 weeks of gestational age, (c) 26 weeks of gestational age. The cesarean scar is intracervical due to a secondary cesarean delivery in the medical history. stars, cesarean scars

Additionally, the known correlation between the number of prior cesarean deliveries and increasing risk of abnormally invasive placentas shows that with each cesarean section, the endometrium underlying the implantation site is damaged. Moreover, with each cesarean section, the uterine incision is located higher in the uterine wall to avoid bladder injury, and in the next pregnancy the uterine scar becomes increasingly accessible to the implanting embryo 43 Sonographic evaluation of post‐cesarean uterine scars imaged during subsequent pregnancies demonstrated that surgery performed during labor is more likely to occur in the cervix.^98^ Half of the women whose cesarean sections are performed without labor have a detectable cervical scar, suggesting that the incision is created in the myometrium.^43^ Only one recent study showed that in a cesarean section with a dilated cervix >5 cm, a low incision causes a higher incidence of large scar defects at 6‐9 months after delivery. However, these scars at 6‐9 months after the cesarean section do not correlate with the scar or niche that remains at the moment a new implantation occurs. Thus, it can be hypothesized that delaying the cesarean section until the cervix has been effaced, thus enabling the uterine incision to be made through thinner myometrium and as lowly as possible, enables a surgeon to make the uterine incision in the cervix rather than in the isthmus of the uterus. An uterine incision through a thinner myometrium may stimulate the uterine recovery and an incision as low as the cervix may be beneficial for subsequent pregnancies, since it has the potential to “hide” the scar from the implanting embryo, reducing the risk of placental abnormalities.^43^, ^95^ As shown in Figure 4A,B, the cesarean scar is intrauterine or intracervical, depending on whether the cesarean section was planned or was an emergency cesarean section, respectively.

## 5 CONCLUDING REMARKS

Placental abnormalities in location and anatomy are low‐lying placentas, placenta previa and abnormally invasive placentas. Placental formation already starts at implantation, at which phase apposition, attachment and invasion occur. It is during this phase that placental abnormalities in location and anatomy come into existence. For the optimal risk stratification of pregnant women and diagnosis and treatment of placental abnormalities in location and anatomy, understanding of the placental structure and development is essential. Reducing the incidence and eventually preventing these placental abnormalities is an important obstetric goal, for which it is imperative that the development and risk factors of these placental abnormalities are studied further and in more detail.

## 6 CLINICAL IMPLICATIONS

Risk stratification of placental abnormalities in location and anatomy is important in all pregnant woman. Preferably, placental localization and anatomy are evaluated at the first or second trimester anomaly scan. When this is not possible, it should at least be performed in high‐risk women. Risk factors for placental abnormalities are well known but understanding the structure and development of the placenta is essential to comprehend the causative relations between these risk factors and placental abnormalities. The most important risk factor for placental abnormalities in location and anatomy is a previous cesarean section. Due to the increasing incidence of cesarean deliveries, the incidence of the abnormalities is rising. Thus, focus on prevention of placental abnormalities, means focus on prevention of the first cesarean section. It is an important task for obstetricians to reduce the number of unnecessary cesarean sections. When a first cesarean section cannot be prevented in a particular clinical situation, delaying the first cesarean section until the cervix is effaced may be an option to reduce placental problems in subsequent pregnancies. It has been shown that a planned cesarean section is associated with a higher risk of a uterine rupture and postpartum hemorrhage compared with an emergency cesarean section in a subsequent pregnancy.^99^, ^100^ In the absence of labor, the uterine incision is made in the uterus rather than the cervix, which also affects the localization of the uterine scar and the healing of the lower uterine segment after cesarean delivery (Figure 4).^101^ So, delaying the first cesarean section until the cervix is effaced may be a successful option to investigate further.

To improve maternal and neonatal outcome, and to prevent placental abnormalities in subsequent pregnancies, it is of utmost importance to encourage women to deliver vaginally if possible.
